# National Update on Measles Cases and Outbreaks — United States, January 1–October 1, 2019

**DOI:** 10.15585/mmwr.mm6840e2

**Published:** 2019-10-11

**Authors:** Manisha Patel, Adria D. Lee, Nakia S. Clemmons, Susan B. Redd, Sarah Poser, Debra Blog, Jane R. Zucker, Jessica Leung, Ruth Link-Gelles, Huong Pham, Robert J. Arciuolo, Elizabeth Rausch-Phung, Bettina Bankamp, Paul A. Rota, Cindy M. Weinbaum, Paul A. Gastañaduy

**Affiliations:** ^1^Division of Viral Diseases, National Center for Immunization and Respiratory Diseases, CDC; ^2^New York State Department of Health; ^3^New York City Department of Health and Mental Hygiene; ^4^Immunization Services Division, National Center for Immunization and Respiratory Diseases, CDC.

*On October 4, 2019, this report was posted online as an *MMWR *Early Release.*

During January 1–October 1, 2019, a total of 1,249 measles cases and 22 measles outbreaks were reported in the United States. This represents the most U.S. cases reported in a single year since 1992 (*1*), and the second highest number of reported outbreaks annually since measles was declared eliminated* in the United States in 2000 (*2*). Measles is an acute febrile rash illness with an attack rate of approximately 90% in susceptible household contacts (*3*). Domestic outbreaks can occur when travelers contract measles outside the United States and subsequently transmit infection to unvaccinated persons they expose in the United States. Among the 1,249 measles cases reported in 2019, 1,163 (93%) were associated with the 22 outbreaks, 1,107 (89%) were in patients who were unvaccinated or had an unknown vaccination status, and 119 (10%) measles patients were hospitalized. Closely related outbreaks in New York City (NYC) and New York State (NYS; excluding NYC), with ongoing transmission for nearly 1 year in large and close-knit Orthodox Jewish communities, accounted for 934 (75%) cases during 2019 and threatened the elimination status of measles in the United States. Robust responses in NYC and NYS were effective in controlling transmission before the 1-year mark; however, continued vigilance for additional cases within these communities is essential to determine whether elimination has been sustained. Collaboration between public health authorities and undervaccinated communities is important for preventing outbreaks and limiting transmission. The combination of maintenance of high national vaccination coverage with measles, mumps, and rubella vaccine (MMR) and rapid implementation of measles control measures remains the cornerstone for preventing widespread measles transmission (*4*).

Measles cases are classified according to the Council of State and Territorial Epidemiologists’ case definition for measles (*5*). Cases are considered internationally imported if at least part of the exposure period (7–21 days before rash onset) occurred outside the United States and rash occurred within 21 days of entry into the United States, with no known exposure to measles in the United States during the exposure period. An outbreak of measles is defined as a chain of transmission of three or more cases linked in time and place as determined by local and state health department investigations.

During January 1–October 1, 2019, a total of 1,249 measles cases were reported in 31 states and New York City,^†^ including 1,211 (97%) among U.S. residents. Median patient age was 6 years (interquartile range [IQR] = 2–22 years); 13% were infants aged <12 months (not routinely recommended to receive MMR vaccine), 31% were children aged 1–4 years, 27% were school-aged children aged 5–17 years, and 29% were adults aged ≥18 years (Table). Among all measles patients, 1,107 (89%) were unvaccinated or vaccination status was unknown, and 142 (11%) had received ≥1 MMR vaccination. Most cases (1,054, 84%) were laboratory-confirmed; among 714 (57%) cases for which specimens were available for molecular sequencing, genotypes B3 (49, 7%) and D8 (665, 93%) were identified. Overall, 119 (10%) patients were hospitalized (median age 6 years, IQR = 1–33 years; 20% were infants aged <12 months), 60 (5%) had pneumonia, and one (0.1%) had encephalitis; no deaths were reported to CDC. Eighty-one cases were imported from other countries^§^ including 52 (64%) cases in U.S. residents returning from travel abroad. Among these 81 internationally imported measles cases, 73 (90%) were in unvaccinated persons or persons for whom vaccination status was unknown.

**TABLE:** Number and vaccination status of measles cases, by age group — United States, January 1– October 1, 2019

Age group	Measles cases no. (%)	Vaccination status no. (%)*		
		Unvaccinated	Vaccinated	Unknown
0–5 mos	43 (3)	43 (100)	0 (0)	0 (0)
6–11 mos	116 (9)	110 (95)	5 (4)	1 (1)
12–15 mos	118 (9)	106 (90)	12 (10)	0 (0)
16 mos–4 yrs	274 (22)	238 (87)	33 (12)	3 (1)
5–17 yrs	339 (27)	295 (87)	26 (8)	18 (5)
18–29 yrs	144 (12)	49 (34)	41 (28)	54 (38)
30–49 yrs	160 (13)	25 (16)	22 (14)	113 (71)
≥50 yrs	55 (4)	6 (11)	3 (5)	46 (84)
**Overall**	**1,249**	**872 (70)**	**142 (11)**	**235 (19)**

* Received ≥1 dose of measles, mumps, and rubella vaccine.

In 2019, 22 outbreaks occurred in 17 states (seven were multistate outbreaks); outbreaks accounted for 1,163 (93%) of all reported cases. Eight outbreaks that occurred in underimmunized, close-knit communities accounted for 85% of all cases; outbreaks associated with NYS and NYC accounted for 934 (75%) of all cases. The median outbreak size and duration were six cases (range = 3–646 cases) and 27.5 days (range = 5–230 days), respectively. The median age of patients with outbreak-related cases was 6 years (IQR = 2–19 years). Most outbreak-related cases occurred in persons who were unvaccinated, or in those for whom vaccination status was unknown (1,032, 89%). Most (57, 70%) of the 81 internationally imported cases were not associated with outbreaks.

Beginning in late 2018, two closely related outbreaks within Orthodox Jewish communities were reported in NYC and NYS. The first began in NYC with an internationally imported case in a returning U.S. traveler on September 30, 2018; this outbreak lasted 9.5 months and included 702 cases. The second outbreak, which began in NYS with an internationally imported case in a foreign visitor on October 1, 2018, lasted 10.5 months and included 412 cases. The NYC outbreak included 53 cases reported by four other jurisdictions, and the NYS outbreak included four cases reported by two other jurisdictions. Among the 1,487 cases reported to CDC during September 30, 2018–October 1, 2019, 1,397 (94%) cases were associated with 26 outbreaks, and 1,114 (75%) were related to outbreaks in NYC and NYS (Figure). Compared with the NYC and NYS outbreaks, the 24 other U.S. outbreaks reported during the same period were of smaller sizes (median = six cases; range = 3–79 cases), and shorter durations (median = 27 days; range = 5–82 days). Median age was similar between the NYC (median = 4 years; IQR = 1–14 years) and NYS (median = 5 years; IQR = 2–14 years) outbreaks, but lower than that in the other U.S. outbreaks (median = 19 years; IQR = 8–25 years). The proportion of unvaccinated patients and patients with unknown vaccination status was similar in NYC (89%), NYS (91%), and other U.S. (87%) outbreaks. The NYC and NYS outbreaks were associated with multiple internationally imported cases (eight in NYC and 10 in NYS), whereas the other U.S. outbreaks were associated with a median of one internationally imported case.

**FIGURE:**
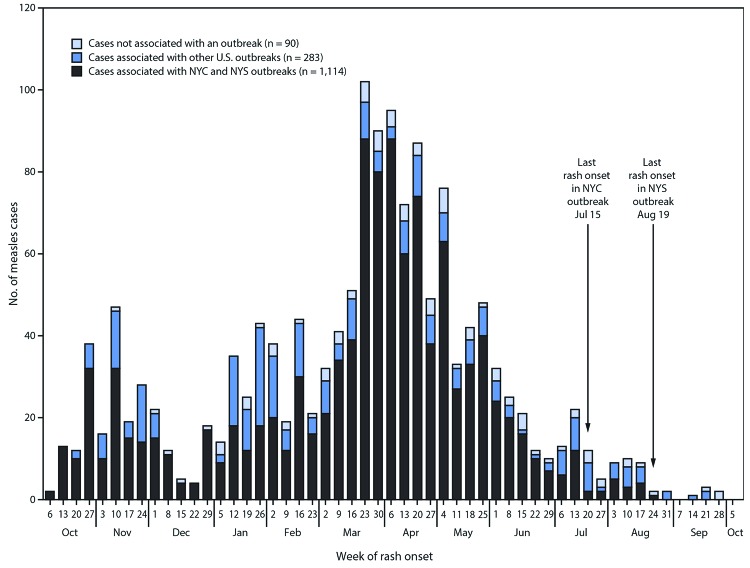
Number of reported measles cases (N = 1,487), by week of rash onset — United States, September 30, 2018–October 1, 2019 **Abbreviations:** NYC = New York City; NYS = New York State.

## Discussion

A total of 1,249 measles cases have been reported in the United States in 2019, with most cases associated with large and closely related outbreaks in New York City and the rest of New York State. Consistent with previous outbreaks that have occurred since measles was declared eliminated in the United States in 2000, most of the other U.S. outbreaks reported in 2019 were of limited size and duration because of high population immunity and rapid implementation of outbreak control measures by local and state public health authorities. In contrast, the two sustained outbreaks in NYC and NYS were larger and lasted longer because of a combination of three important risk factors for measles transmission: 1) pockets of low vaccination coverage and variable vaccine acceptance; 2) relatively high population density and closed social nature of the affected community; and 3) repeated importations of measles cases among unvaccinated persons traveling internationally and returning to or visiting the affected communities. These two almost year-long outbreaks placed the United States at risk for losing measles elimination status. Robust responses in NYC and NYS with multiple partners involved vaccination efforts, including administration of approximately 60,000 MMR vaccine doses in the affected communities; tailored communication campaigns; partnerships with religious leaders, local physicians, health centers, and advocacy groups; and use of local public health statutory authorities. These efforts ended transmission before the 12-month elimination deadline, with the most recent cases reported with rash onset on July 15, 2019, in NYC and August 19, 2019, in the rest of NYS. Both jurisdictions have since passed two incubation periods for measles with no additional reported cases associated with these outbreaks as of October 1, 2019; however, continued vigilance is important to ensure that elimination is sustained. 

Increased global measles activity and existence of undervaccinated communities place the United States at continual risk for measles cases and outbreaks (*6*). Control measures for measles outbreaks have been in place for decades in the United States to limit transmission and prevent reestablishment of endemic transmission (*7*,*8*). Core elements include a highly sensitive surveillance system with multiple feedback loops between providers, laboratories, local and state public health authorities, and CDC. These measures are coupled with rapid activation of local and state public health departments in response to every measles case to determine the source of infection, identify susceptible contacts, and implement control measures, including postexposure prophylaxis, exclusion and quarantine, and community-wide vaccination. High national MMR vaccination coverage remains the foundation for preventing more widespread measles transmission (*9*). The limited size and duration of 24 of the 26 outbreaks reported during September 2018–September 2019 indicate that high baseline vaccination coverage and standard measles control measures effectively controlled most outbreaks in the United States.

Measles outbreaks in undervaccinated, close-knit communities pose challenges that require considerations beyond standard control measures. To identify and protect communities, routine assessments, including school audits and use of electronic immunization information systems to ascertain local vaccination coverage and vaccine access, could help identify critical gaps and resource needs. Because health-seeking behaviors in members of close-knit communities are routinely informed by discussions with like-minded community members, establishing strong community partnerships before outbreaks occur can foster overarching goals to protect the community against public health threats. Public health authorities might also benefit from identifying trusted community liaisons who can assist with case and contact investigations so that standard control measures can be rapidly implemented.

Undervaccinated, close-knit communities are not unique to the United States and exist around the world. These communities are at high risk for outbreaks of vaccine-preventable diseases, which threaten the health and safety of vulnerable persons within, as well as outside of, these communities. Therefore, public health authorities need to identify pockets of undervaccinated persons to prevent these outbreaks, which require substantial resources to control. A preventive strategy to build vaccine confidence is important, especially one that uses culturally appropriate communication strategies to offset misinformation and disseminate accurate information about the safety and importance of vaccination in advance of outbreaks.

**Summary**

**What is already known about this topic?**

Measles was eliminated in the United States in 2000. High national coverage with measles, mumps, and rubella vaccine and rapid implementation of measles control measures prevent widespread measles transmission.

**What is added by this report?**

During January–September 2019, 1,249 U.S. measles cases were reported, the highest annual number since 1992. Eighty-nine percent of measles patients were unvaccinated or had an unknown vaccination status, and 10% were hospitalized. Eighty-six percent of cases were associated with outbreaks in underimmunized, close-knit communities, including two outbreaks in New York Orthodox Jewish communities that threatened measles elimination status in the United States.

**What are the implications for public health practice?**

Ensuring high rates of measles immunization in all communities is critical to sustaining measles elimination.

## Footnotes

* According to the World Health Organization, measles elimination status is based on the absence of endemic measles transmission in a defined geographical area (e.g., region or country) for ≥12 months in the presence of a well-performing surveillance system. https://www.who.int/immunization/policy/position_papers/measles/en/.
